# Influence of 2′-Fucosyllactose on the Microbiota Composition and Metabolic Activity of Fecal Cultures from Breastfed and Formula-Fed Infants at Two Months of Age

**DOI:** 10.3390/microorganisms9071478

**Published:** 2021-07-09

**Authors:** Alicja M. Nogacka, Silvia Arboleya, Naghmeh Nikpoor, Jeremie Auger, Nuria Salazar, Isabel Cuesta, Laura Mantecón, Gonzalo Solís, Miguel Gueimonde, Thomas A. Tompkins, Clara G. de los Reyes-Gavilán

**Affiliations:** 1 Department of Microbiology and Biochemistry of Dairy Products, Instituto de Productos Lácteos de Asturias (IPLA-CSIC), 33300 Villaviciosa, Asturias, Spain; alicja.nogacka@ipla.csic.es (A.M.N.); silvia.arboleya@ipla.csic.es (S.A.); nuriasg@ipla.csic.es (N.S.); icuesta@ipla.csic.es (I.C.); mgueimonde@ipla.csic.es (M.G.); 2 Institute of Health Research of the Principality of Asturias (ISPA), 33011 Oviedo, Asturias, Spain; Laura_mantecon@hotmail.com (L.M.); GSOLIS@telefonica.net (G.S.); 3 Rosell® Institute for Microbiome and Probiotics, Montreal, QC H4P 2R2, Canada; nnikpoor@lallemand.com (N.N.); jeremieauger@gmail.com (J.A.); ttompkins@lallemand.com (T.A.T.); 4 Pediatrics Service, Central University Hospital of Asturias (HUCA-SESPA), 33011 Oviedo, Asturias, Spain

**Keywords:** human milk oligosaccharides, 2′-fucosyllactose, microbiota, in vitro model, infants, breastfeeding, *Bifidobacterium*

## Abstract

Although breast milk is considered the gold standard of nutrition for infant feeding, some circumstances may make breastfeeding difficult. Several commercial milk preparations include synthetic human milk oligosaccharides (HMOs) in their composition. However, the effect of HMOs on the establishment of the intestinal microbiota remains incompletely understood. Independent batch fermentations were performed with feces from six full-term infant donors of two months of age (three breastfed and three formula-fed, exclusively) in the presence of 2′fucosyllactose (2′FL), one of the most abundant HMOs in human milk. Microbiota composition was analyzed by 16S rRNA gene sequencing at baseline and at 24 h of incubation. The 2′FL consumption, gas accumulation, and levels of different metabolites were determined by chromatography. Microbiota profiles at baseline were clearly influenced by the mode of feeding and by the intrinsic ability of microbiotas to degrade 2′FL. The 2′FL degradation rate clustered fecal cultures into slow and fast degraders, regardless of feeding type, this being a determinant factor influencing the evolution of the microbiota during incubation, although the low number of donors precludes drawing sound conclusions. More studies are needed to decipher the extent to which the early intervention with HMOs could influence the microbiota as a function of its ability to utilize 2′FL.

## 1. Introduction

Microbial colonization of the gut starts immediately after birth and provides a stimulus for the development of the intestine, the modulation of the immune system, and the physiological homeostasis of the neonate [1]. The gut microbiota in early life evolves continuously and is influenced by several factors such as the mode of feeding (mother’s milk vs. formula milk). Human milk oligosaccharides (HMOs) are a group of fucosylated, sialylated, and neutral oligosaccharides with diverse structures, which are abundant in human milk [2,3]. Lactating women differentially produce these molecules, and their production also varies with lactation [4,5]. HMOs contribute to the correct establishment of the intestinal microbiota in neonates [6]. These molecules are resistant to hydrolysis by intestinal enzymes and reach the colon where they can be metabolized by the intestinal microbiota or can be excreted undegraded with feces. These oligosaccharides can act as prebiotics, stimulating the growth of beneficial bacteria, such as bifidobacteria [7]. The intestinal fermentation of HMOs can result in the production of short-chain fatty acids (SCFA), some organic acids and ethanol [8]. Moreover, HMOs mimic the structure of certain glycans present in the surface of host’s epithelial cells and can act as receptors for pathogens, thus preventing their adhesion to host’s cells [1,9]. HMOs also contribute to modulate the immune system and to improve the intestinal barrier function [10].

The ability of mothers to produce HMOs is conditioned by genetic factors, namely the presence of active forms of genes *FUT*2 and *FUT*3 that encode two different fucosyltransferases affecting the secretion status and Lewis blood group antigens [9,11]. Most mothers have an active *FUT*2 gene (secretor status) for fucosyltransferase, an enzyme responsible for adding fucose by α1,2 linkage to terminal galactose to form α1,2-fucosylated oligosaccharide structures. In milk of mothers with secretor status, 2′ fucosyllactose (2′FL) and lacto-N-fucopentaose I are the most abundant HMOs, and the concentration of 2′FL is higher in the early stages of lactation, especially during the first month of life [10]. However, mothers with non-secretor status (about 20%) do not produce these oligosaccharides [9]. Currently, the later health consequences of a low exposure to HMOs in early life are probably underestimated [9]. In this sense, 2′FL is being prepared and commercialized by several companies to be added to infant foods.

HMOs are able to promote the selective growth of some members of the genus *Bifidobacterium,* as well as some *Bacteroides* species [12,13,14], whereas lactobacilli generally have a limited ability to degrade HMOs [14,15,16]. The ability of bifidobacteria to utilize HMOs as fermentable substrate for growth provides these microorganisms with a competitive advantage to colonize and persist in the intestine of babies fed mother’s milk [17]. The species *Bifidobacterium bifidum*, *Bifidobacterium longum* subsp. *infantis* and *B. longum* subsp. *longum* are specially adapted to metabolize HMOs. Their genomes are enriched in genes encoding glycosyl hydrolases, serving as gut forager of both host and diet-derived glycans [1,16,18] and playing a key role in the establishment of the gut microbiota in early life [19].

As some circumstances require the use of formula instead of breast milk, a wide variety of commercial milk formulations are currently available, which include HMOs and/or probiotics and that intend to mimic human breast milk. Hence, there is a need to better characterize the effects of HMOs on the microbiota of newborns. The aim of the present study was to determine the effect of 2′FL on the fecal microbiota composition using fecal cultures of full-term vaginally delivered newborns of two months of age fed exclusively with breastmilk or formula.

## 2. Materials and Methods

### 2.1. 2′-Fucosyllactose Commercial Preparations

Three commercial preparations of 2′FL were used: a 2′FL preparation from DuPont Nutrition and Health, Aequival^®^ 2′-FL (Friesland Campina Ingredients, Paramus, NJ, USA), and 2′-FL from Jennewein Biotechnologie GmbH (Rheinbreitbach, Rheinland-Pfalz, Germany) hereafter referred to as 2′FL-A, 2′FL-B and 2′FL-C, respectively. The three preparations were freshly diluted each week in MilliQ water at 30% (*w/v*) and sterilized by filtration through a pore size of 0.45 µm.

### 2.2. Fecal Sample Collection

Fecal samples were obtained from six vaginally delivered full-term healthy children at two months of age. Three newborns were exclusively fed with breastmilk (BF) of their mothers and the other three received exclusively formula milk (FF). The infant named FF1 received the formula Nutriben Innova 1^®^ (Alter, Madrid, Spain) that included a *Bifidobacterium animalis* subsp. *lactis* strain as probiotic, and the HMOs 2′FL and Lacto-*N*-neotetraose. Infants FF2 and FF3 were fed with the formula Blemil plus 1 forte (Ordesa, Huesca, Spain) that included long-chain and short-chain fructooligosaccharides as prebiotics. Infants were recruited at the Neonatology Unit of the Pediatrics’ Service at the Central University Hospital of Asturias (HUCA, Northern Spain). All infants were discharged from the Hospital at 2–3 days of life. The Regional Ethical Committee of Asturias Public Health Service (n º 236/19) approved the study, and informed written consent was obtained from each infant’s parents. Samples were collected at home over one or two weeks from nappies immediately after defecation using a sterile spatula and were frozen in sterile tubes at −20 °C until their transportation to the laboratory within a week from collection. In an anaerobic chamber, a 1/5 (*w/v*) dilution of a minimum of 20 g of fecal samples, obtained by mixing consecutive samples from the same infant, was carried out in pre-reduced PBS solution with 25% (*v/v*) glycerol, vortexed for 10 min and stored in 20–30 mL aliquots at −80 °C until use.

### 2.3. Fecal Batch Culture Fermentation

Independent pH-uncontrolled fecal batch fermentations from the different infant donors were performed in a basal medium for cultivation of infant feces (BMIF) added with 5% (*v/v*) of reconstituted formula milk (Novalac, initiation formula 0–6 months, Paris, France) as described by Arboleya et al. [20]. The three 2′FL commercial preparations, named as 2′FL-A, 2′FL-B and 2′FL-C, and a negative control with no 2′FL added, were included.

BMIF medium was pre-reduced overnight in anaerobic conditions one day before the batch fermentation experiments. On the day of the assay, the stocks of fecal samples were thawed in anaerobic conditions, centrifuged, washed, and re-suspended in pre-reduced sterile PBS to a concentration of 1/10 (*v/v*). Pre-reduced BMIF medium was inoculated (10% *v/v*) with the fecal homogenate and distributed into bottles of the ANKOM RF system (Ankom Technology, Macedon, NY, USA) to a final volume of 80 mL per bottle. The fecal cultures were allowed to stabilize 4 h at 37 °C in anaerobiosis. Then, the different 2′FL commercial preparations were added to the corresponding bottles at a final concentration of 0.2% (*v/v*), plus formula milk, and vitamins at the concentrations required (basal conditions). Bottles with the different 2′FL preparations and the negative control, were incubated under anaerobiosis at 37 °C for 24 h. Samples (1 mL) were taken in duplicate at time 0 before incubation (basal conditions: baseline) and after 24 h of incubation. The samples were centrifuged at full speed for 15 min, and pellets and supernatants were stored separately at −20 °C until analyses.

### 2.4. Microbiota Composition Analysis

The bacterial taxonomic composition of the pellets from the fecal batch cultures was assessed by 16S rRNA gene sequencing of the V3–V4 region. In brief, the QIAamp Fast DNA Stool Mini Kit (Qiagen; Düsseldorf, Germany) was used for genomic DNA extraction as per manufacturer’s protocol, with some modifications, as indicated next. Pellet from 1 mL of fecal culture was first washed with PBS (VWR Chemicals, Solon, OH, USA) and centrifuged at maximum speed at 4 °C. The step of cellular lysis was performed at 95 °C for 10 min. The digestion with proteinase K was done for a total of 800 µL of lysate, maintaining the proportion of proteinase K and buffer AL as described by the manufacturer’s protocol. DNA was eluted in 60 µL of buffer ATE. DNA concentrations were determined using a Nanodrop Spectrophotometer (ND-1000; V3.8.1 program, Waltham, MA, USA). Samples (48) were processed for amplicon sequencing of the 16S rRNA V3-V4 regions. Extracted genomic DNA samples (50 ng) were amplified using 1X KAPA HiFi HotStart ReadyMix (Roche, cat # KK2802) with 200 nM of universal 16S primers (forward 5′-CCTACGGGNGGCWGCAG-3′ and reverse 5′-GACTACHVGGGTATCTAATCC-3′) in a 25 µL reaction volume [21]. Cycling conditions for the PCR amplicon were as follows: initial denaturation at 95 °C for 3 min followed by 25 cycles of 95 °C for 30 s, primer annealing at 55 °C for 30 s and primer extension at 72 °C for 30 s before a 5 min final extension at 72 °C. Commercially available 20 strains mock communities (ATCC, cat # MSA-1002-even mix and MSA-1003-staggered mix) were used as sequencing controls and water as no template amplification control. PCR products were visualized on a 2% agarose precast E-Gel stained with SYBR Safe dye (Invitrogen cat # G72080). Amplicons were purified with Agencourt AMPure beads (Beckman Coulter, cat # A63881) following Illumina 16S Metagenomic sequencing library preparation’s protocol. A second round of amplification using 5 µL of the purified amplicon PCR reaction as template was performed in 25 µL reactions containing 2.5 µL of each of the Nextera XT V2 primers sets A, B and C (Illumina, cat # FC-131-2001, FC-131-2002 and FC-131-2003) and 1X KAPA HiFi ReadyMix. The same cycling conditions as the Amplicon PCR were used, except that only 8 cycles were necessary to attach the different index combinations used as tags to identify each sample during multiplexing. PCR reactions were again purified with AMPure beads before individual fluorescent quantification by Quant-iT PicoGreen dsDNA assay (Life Technologies, cat # P7589) with a Varioskan LUX microplate reader (ThermoScientific, cat # VL0000D0) with 485 nm excitation and 520 nm emission wavelengths. Volumes corresponding to 200 ng of each purified Index PCR reaction were pooled using an EpMotion 5075 liquid handling robot (Eppendorf) and this pool was quantified with QuBit Broad Range assay (ThermoScientific, cat # Q32853; Waltam, MA, USA) following manufacturer’s instructions. Finally, the diluted pool (amplicon size 599 bps) and PhiX mixture was loaded in a MiSeq Reagent 600 cycles v3 Kit cartridge (Illumina, cat # MS-102-3003) and processed for 2 × 300 cycles paired-end sequencing on a MiSeq instrument.

### 2.5. Gas and pH Monitorization

The pH of fecal cultures was determined at time 0 and after 24 h of incubation with a pHmeter SensION + PH3 (HACH, Barcelona, Spain).

The cumulative gas produced during the different fermentation conditions was monitored in real-time by using the ANKOM RF system as indicated by Nogacka et al. [22]. This system provides the increases in pressure (psi) that can be converted to mL of gas produced, using the Ideal Gas Equation:

(1) V=Vj×Ppsi×0.068004084

where: *V* = gas volume at 39 °C in mL, *Vj* = headspace of digestion gas bottle in mL, *Ppsi* = cumulative pressure recorded by the Gas Monitor System software.

### 2.6. Analysis of 2′Fucosyllactose, Lactose, Monosaccharides and Organic Acids by HPLC

The consumption of 2′FL, variations in the levels of lactose and the monosaccharides glucose, galactose and fucose, as well as organic acids formed during incubation (lactic, pyruvic, succinic, and formic) were analyzed by HPLC. Standard solutions of fucose (20, 40, 60, 80 y 100 mg/100 mL) and 2′FL-A (60, 120, 180, 240, and 300 mg/mL) were prepared in BMIF medium. Cell-free supernatants from cultures were filtered (0.45 µm), injected using an Alliance 2695 module injector and separated by ion-exclusion chromatography through a column ICSep ICE-ION (Teknokroma Analitica, Barcelona, Spain). A PDA 966 photodiode array detector was used for the determination and quantification of organic acids, a 2414 differential refractometer detector for determination and quantification of carbohydrates (2′FL, lactose, glucose, fucose and galactose) and the Empower software (Walters, Milford, MA, USA) for identification and quantification of peak areas. Chromatographic and analysis conditions were those described elsewhere [23]. Results were expressed in mg/100 mL. Variations in the levels of the analyzed metabolites at 24 h of incubation were calculated when appropriate for each fermentation batch with respect to the basal conditions (time 0, ∆). The consumption of 2′FL at 24 h of incubation was calculated with respect to the concentration of 2′FL in basal conditions (time 0), considering this as the 100%.

A semi-quantitative determination of the degree of purity of 2′FL was carried out in aqueous solutions at 0.3% and 0.1% (*w/v*) in the same chromatographic conditions indicated above, using the differential refractometer detector, by calculating the percentage of the peak corresponding to 2′FL with respect to the total peak areas obtained at different elution times.

### 2.7. Analysis of Short-Chain Fatty Acids by Gas Chromatography

The analysis of SCFAs was performed by Gas Chromatography (GC) in the fecal culture supernatants to quantify acetic, propionic, butyric acid (major SCFAs), as well as isobutyric and isovaleric acid (branched chain fatty acids: BCFAs). 250 µL of culture supernatants collected at time 0 and 24 h of incubation were mixed with 0.3 mL methanol, 0.05 mL of the internal standard solution (2-ethylbutyric acid 1.05 mg/mL), and 0.05 mL of 20% (*v/v*) formic acid. The mixture was centrifuged, and the supernatant was collected for SCFA quantification in a system composed of a 6890N GC injection module (Agilent Technologies Inc., Palo Alto, Ca, USA) connected to a flame injection detector (FID) and a mass spectrometry (MS) 5973N detector (Agilent), as described previously [24,25]. Samples were analyzed in triplicate and results were expressed in µg/mL. Increments (∆) in the levels of these compounds at 24 h of incubation with respect to the basal conditions (time 0) were calculated for each fermentation batch with the different 2′FL preparations tested.

### 2.8. Microbiome Community—Bioinformatic Data Processing and Statistical Analyses

The fastq files of the 16S sequencing were imported into QIIME2 (Quantitative Insight Into Microbial Ecology–2) as artefacts. These QIIME2 compliant formats allow tracking the transformation of the data step-by-step [26]. First, the quality filter software was used to process the demultiplexed amplicons. The imported data set was inspected, the reads were trimmed at 240 base pairs, and the quality-filter q-score using the default parameters was employed for quality control. The reads were then clustered into amplicon sequence variants (ASVs) with the denoising algorithm Deblur of the QIIME2 suite [27]. The feature classifier was used to attribute the ASVs to the closest known taxa using QIIME2′s sk-learn classification module [28]. The taxonomy file (linking ASV sequences to known taxonomic groups) was trained on a 99% clustered GreenGenes database. The taxonomic profiles, individually or aggregated into the four groups (combination of BF/FF and Fast/Slow 2′FL degraders), were presented as taxonomic stacked bar-plots using QIIME2′s software. The alpha diversity (Shannon index) calculated in QIIME2 was exported for plotting into GraphPad Prism v7.04 software. Alpha diversity values at baseline were compared between fecal inocula from BF and FF donors subclassified by 2′FL degradation velocity using a Kruskal-Wallis test followed by a Dunn’s post-hoc test for pairwise comparisons.

Principal Component Analysis (PCA) plots and Heatmaps were constructed using the web tool ClustVis [29]. PCA of microbial taxonomic compositional data were scaled by Pareto; the standard PCA method of Singular Value Decomposition (SDV) with imputation was used to calculate the principal components. For Heatmap representations at the family taxonomic level, original values were logarithmically transformed, rows were normalized by unit variance scaling and clusters were made by using correlation distance and average linkage. Logarithmic Linear Discriminant Analysis (LDA) Effect Size (LEfSe) [30] was used to estimate at the family taxonomic level the microorganisms differing significantly among each of the 4 groups (combination of BF/FF and Fast/Slow 2′FL degraders), by using a Kruskall–Wallis sum-rank test (alpha significance level of 0.05) and a Wilcoxon test (alpha significance level of 0.05) for pairwise comparison. This was followed by an LDA to estimate the effect-size (threshold of 2) of each differentially abundant feature. Cladograms were used to represent hierarchically the most differentially abundant taxa generated from LEfSe analysis.

A paired sample Student’s *t*-test was used to compare relative abundances between baseline and after 24 h of incubation. The most abundant taxa obtained by 16S rRNA gene sequencing at the family and genus taxonomic levels were compared for the four groups of inocula defined in the study on the basis of the ability to degrade 2′FL and feeding type of donors.

### 2.9. Statistical Analyses of Microbial Metabolites

Statistical analysis of results reflecting the metabolic activity of fecal cultures was performed using the software SPSS v.26 (SPSS Inc., Chicago, IL, USA). Data from 2′FL consumption, pH evolution, gas production, and variations in the levels of lactose, monosaccharides, organic acids, SCFA and BCFA were compared in fecal cultures of infant donors at the end of fermentation (24 h). Data were analyzed according to the rate of 2′FL fermentation (fast and slow), fecal sample donor feeding type (BF vs. FF), or the treatment (comparison among the different 2′FL commercial preparations). The U-Mann Whitney test was carried out to compare cultures from BF and FF infants and from fast and slow fermenter donors, whereas the comparison among cultures added with the different commercial preparations of 2′FL was performed through a Kruskal-Wallis test followed by a Dunn’s post-hoc test for pairwise comparisons.

## 3. Results

### 3.1. 2′-FL Degradation Profile in Fecal Cultures

**Figure 1:**
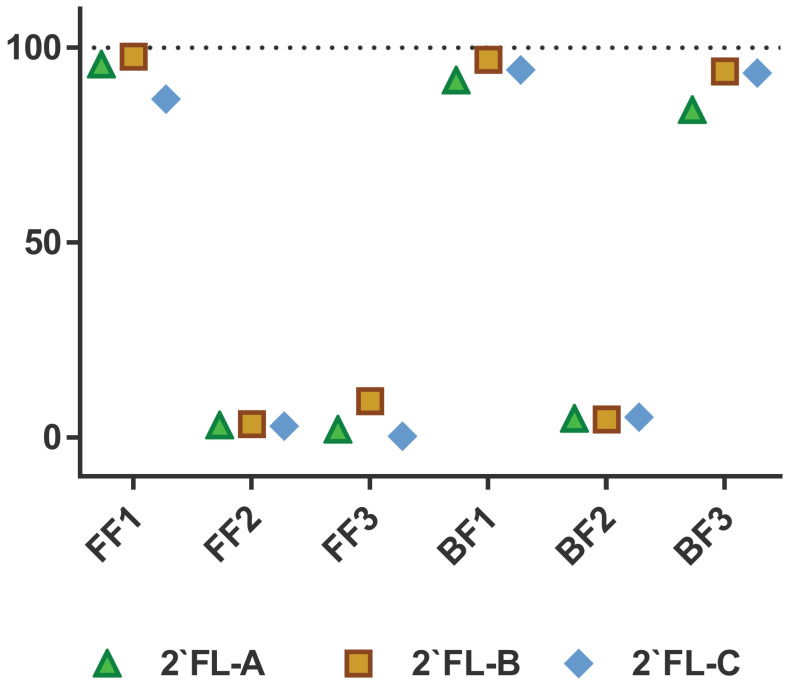
2-′fucosyllactose (2′FL) consumption (% of decrease in the levels of 2′FL from baseline values considered as 100%) by fecal cultures after 24 h of incubation, disaggregated by infant donor and 2′FL commercial preparation. FF: formula-fed fecal infant donor; BF: breastfed fecal infant donor. Numbers indicated the different infant donors. 2′FL-A was prepared by DuPont Nutrition and Health; 2′FL-B was from Friesland Campina (Aequival^®^): 2′FL-C was provided by Jennewein Biotechnologie GmbH.

An individual-based approach evidenced a clear bimodal distribution of the ability to ferment 2′FL by the intestinal microbial communities from the different infants (Figure 1). Thus, whereas at 24 h of incubation fecal cultures from FF1 baby and two BF babies have utilized about 90% of 2′FL present in the medium (fast degraders), fecal cultures of babies FF2 and FF3 and one BF baby have consumed less than 5–6% (slow degraders) (Figure 1). Moreover, a similar fermentation pattern for the three 2′FL commercial preparations was obtained in fecal cultures of each of the different infant donors (Figure 1), indicating no differences in fermentability rates by the microbiota between the three 2′FL commercial products.

### 3.2. Microbiota Profile of Fecal Inocula at Baseline

**Figure 2:**
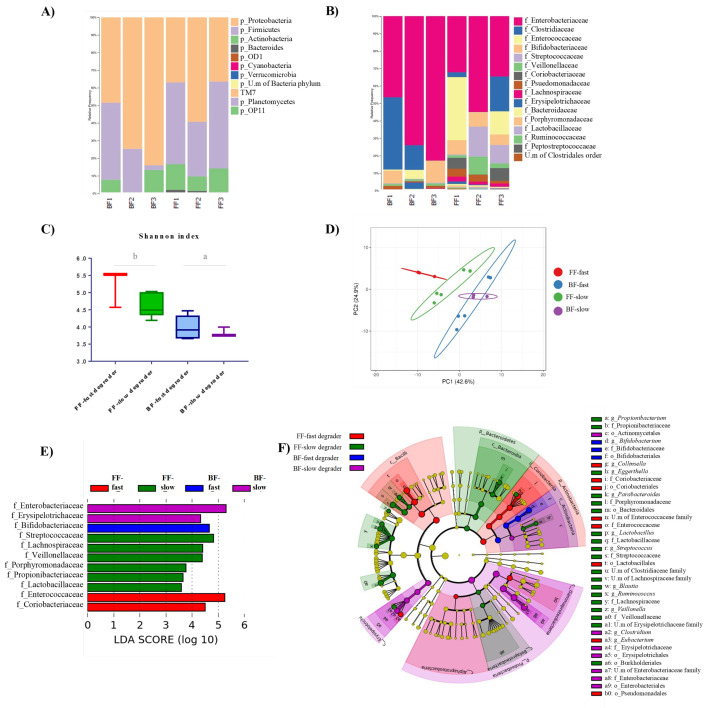
Characteristics of the microbiota from fecal inocula at baseline (T0). Relative abundance of bacteria at Phylum (**A**) and Family levels (**B**). Alpha diversity (Shannon index) of fecal inocula from the four groups generated according to feeding type and 2′FL degradation ability (**C**): letters indicate significant differences (*p* < 0.05) between groups below lines. Principal Component Analysis of microbial compositional taxonomic data of inocula (**D**). Linear Discriminant Analysis (LDA) scores of microbial families significantly altered in the four inocula groups (combination of breastmilk (BF)/formula milk (FF) and Fast/Slow 2′FL degraders) (**E**). The most differentially abundant taxa in BF-fast degraders, BF-slow degrader, FF-fast degrader, and FF-slow degraders are represented as a cladogram generated from Logarithmic Linear Discriminant Analysis (LDA) Effect Size (LEfSe) analysis data where the color intensity of each dot is proportional to its effect size (**F**).

The microbiota composition of fecal samples from the different infant donors was analyzed by 16S rRNA gene profiling. Overall, the major relative abundances of phyla in the six fecal inocula followed the order: Proteobacteria > Firmicutes > Actinobacteria > Bacteroidetes. However, Proteobacteria were more abundant in the inocula from BF infants, whereas Firmicutes were more abundant in the inocula of FF babies (Figure 2A). These trends were confirmed at the family level, Enterobacteriaceae (Proteobacteria) being the most abundant microbial family in all samples, and these microorganisms also being at higher proportion in the inocula from the three BF vs. FF babies. In contrast, the family Veillonellaceae (Firmicutes) was found at higher proportion in the inocula of FF babies, whereas members of the family Bifidobacteriaceae (Actinobacteria) were found at higher proportion in the inocula of BF 2′FL-fast degrader donors (BF1 and BF3) (Figure 2B). An overall heterogeneity was found among the fecal inocula of different donors, highlighting the considerably higher levels of the family Streptococcaceae (Firmicutes) in the inocula of the two FF non-degrader babies (FF2 and FF3), as well as the very low levels of Bifidobacteriaceae in the samples of the only BF non-degrader baby (BF2). BF inocula also displayed lower alpha diversity (Shanon index) than FF samples, and samples from slow degraders presented significantly lower diversity than those from fast degraders in both feeding groups (Figure 2C).

The PCA analysis of the merged datasets of microbiota profiles at baseline (T0) showed that inocula from BF and FF babies clustered separately. However, whereas slow and fast degrader inocula from babies receiving formula clearly distinguished between them, those from breastfed infants clustered more closely with each other (Figure 2D). LEfSe analysis was performed by using the 16S rRNA gene profiling data in order to identify taxa at the family level which were responsible for the differential profiles of the four inocula groups (Figure 2E). The results identified differentially increased abundance of Bifidobacteriaceae in the inocula from BF-fast degraders, and higher abundance of Enterobacteriaceae and Erysipelotrichaceae in samples from the BF-slow degrader donor. Enterococccaceae and Coriobacteriaceae were identified as the most differentially abundant features in the inocula from the FF-fast degrader, whereas Streptococcaceae, Lachnospiraceae, Veillonellaceae, Porphyromonadaceae, Propionibacteriaceae, and Lactobacillaceae were differentially most abundant in samples from FF-slow degraders. The cladogram generated from LEfSe results (Figure 2F) highlights the differential abundance of several taxons in the groups, as it is the case of the genus Bifidobacterium in BF-fast degrader samples, the genus Clostridium in those from the BF-slow degrader, Eubacterium and Collinsella in samples from the FF-fast degrader, and Parabacteroides, Veillonella, Blautia and Lactobacillus in the inocula from FF-slow degraders. All these data evidenced that the four groups of fecal inocula were characterized by clearly differentiated microbiota profiles at baseline.

### 3.3. Microbiota Evolution during Incubation of Fecal Inocula in the Presence of 2′FL

**Figure 3:**
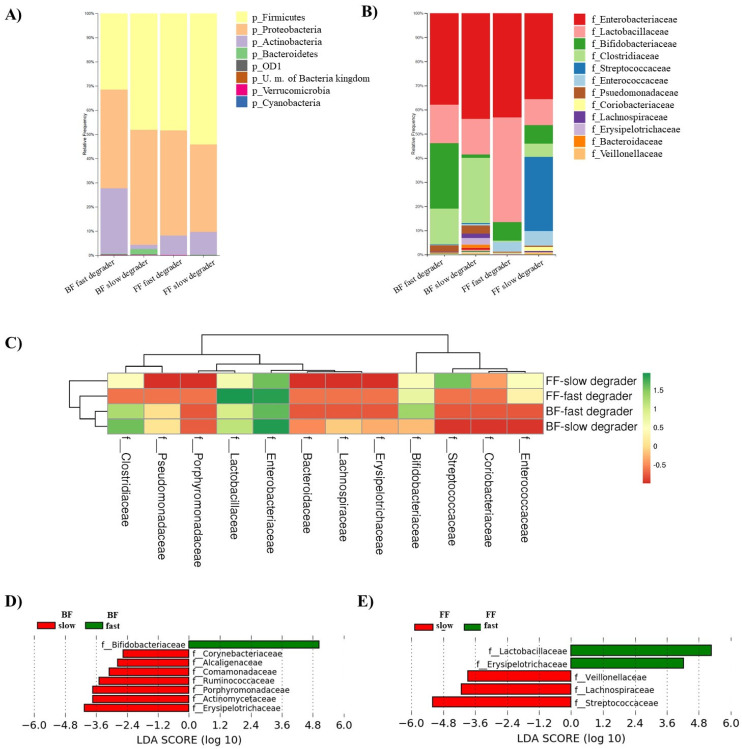
Characteristics of the microbiota from fecal cultures after 24 h of incubation in the presence of 2′FL in the four groups considered according to feeding type and 2′FL degradation rate: BF-fast degraders, BF-slow degrader, FF-fast degrader, FF-slow degraders. Relative abundance of bacteria at Phylum (**A**) and Family levels (**B**). Heatmap of the log-transformed relative abundances of microbial taxa at the family level, which display differences in microbial profiles among the four culture groups (combination of BF/FF and Fast/Slow 2′FL degraders) (**C**). Linear Discriminant Analysis (LDA) scores of microbial taxa at family level that were differentially altered in cultures of fast (green) and slow (red) degrades, according to the feeding type of fecal donors: breastfed (**D**) or formula-fed (**E**).

Different microbiota composition profiles were observed after incubation of fecal cultures in the presence of 2′FL (Figure 3A,B). Firmicutes and Proteobacteria became the most abundant phyla, followed by Actinobacteria (Figure 3A). Lactobacillaceae family mainly accounted for Firmicutes levels in all groups and Streptococcaceae also did in cultures of FF-slow degrader donors (Figure 3B). In cultures of the FF-fast degrader baby, Lactobacillaceae became predominant and more abundant than in fecal cultures from the other donors. Clostridiaceae displayed higher relative abundances in fecal cultures of BF than in those of FF donors. Enterobacteriaceae remained the most abundant in all cultures except in those corresponding to the FF-fast degrader donor. Bifidobacteriaceae attained higher abundances in cultures of BF-fast degrader donors, intermediate levels in cultures of FF donors (both fast and slow degraders) and remained at low levels in the culture of the BF-slow degrader infant donor (Figure 3C).

LEfSe analysis was performed separately for cultures of BF and FF donors in order to identify the main families responsible for the differential microbial profiles associated with the slow and fast degradation of 2′FL (Figure 3D,E). In cultures from BF infants, the family Bifidobacteriaceae for fast degraders and members of Erysipelotrichaceae, Actinomycetaceae, Porphyromonadaceae, Ruminococcaceae, Comamonadaceae, Alcaligenaceae, and Corynebacteriaceae for slow degraders were the most differentially abundant. In contrast, in cultures from FF donors, Lactobacillaceae and Erysipelotrichaceae families for fast degraders and Streptococcaceae, Lachnospiraceae, and Veillonellaceae members for slow degraders marked the differential profiles associated with the velocity of degradation of 2′FL.

Looking for shifts occurring on the microbial groups in fecal cultures during incubation with 2′FL, we examined variations of the most abundant microbial taxa (relative abundance >1% in any of the samples) at 24 h of incubation with respect to baseline (T0) (Table 1). The most relevant finding in fecal cultures of BF 2′FL-fast degrader donors was the pronounced and significant increase in bifidobacteria, which was accompanied by a decrease in Enterobacteriaceae and streptococci and by slight or no variations in other less abundant microoorganisms, with the exception of the significant decrease in Veillonella. In contrast, the only BF 2′FL-slow degrader donor displayed very low bifidobacteria levels and a marginal increase, with no other significant change but a significant decrease in Enterobacteriaceae. An abrupt and significant increase in lactobacilli occurred in fecal cultures of the only FF 2′FL-fast degrader donor, whereas a significant decrease of Enterococcaceae and streptococci was observed. No noticeable variations were observed in bifidobacteria and the abundance of most of the minor microbial groups decreased, only reaching statistical significance for the family Erysipelotrichaceae and genera Veillonella and Lachnospira. The most notable change in fecal cultures of FF 2′FL-slow degraders was the significant increase in the Streptococcus genus whereas bifidobacteria levels did not change significantly. Significant decreases were also found in these cultures for Enterobacteriaceae as well as for other less abundant groups such as Pseudomonas, Veillonella, Parabacteroides, Blautia, and members of the Ruminoccocaceae family.

### 3.4. Microbial Metabolism

#### 3.4.1. Effect of 2′FL Commercial Preparations on the Metabolite Profile of Fecal Cultures

**Figure 4:**
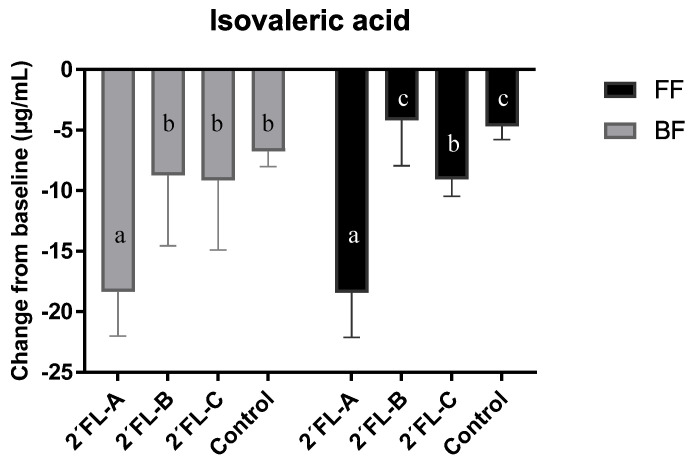
Variations in the levels of isovaleric acid with respect to values in baseline (time 0) after 24 h of incubation in fecal cultures of three BF and three FF infant donors. Different letters in bars indicate significant differences (*p* < 0.05) among the three 2′FL commercial preparations and controls analyzed separately in cultures of FF and BF infants.

The chromatographic analysis of the three 2′FL commercial preparations revealed different degrees of purity for this carbohydrate: 93% in the product A, 87.1% in product B, and 87.8% in product C. The metabolite profiles of the fecal cultures supplemented with each 2′FL preparations were similar for all metabolites tested after 24 h of incubation (data not shown), except for isovaleric acid (Figure 4). Concentrations of isovaleric acid decreased over the 24 h incubation in all commercial preparations tested compared to baseline levels. However, the decrease was significantly more pronounced with 2′FL-A, which had the highest content of 2′FL compared with the other commercial preparations and control (with no 2′FL added), both in fecal cultures of FF and BF donors.

#### 3.4.2. Effect of Donor Feeding Type on the Metabolic Activity of Fecal Cultures

Gas production, pH, microbial SCFA (acetic, propionic and butyric acids), BCFA (isobutyric and isovaleric acids), lactic acid, pyruvic acid, succinic acid, and formic acid, were measured as indicators of the fecal cultures’ metabolic activity.

**Figure 5:**
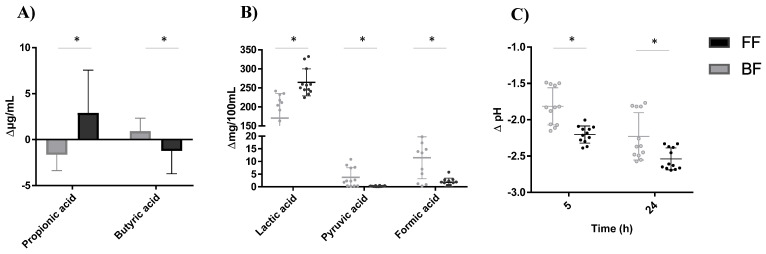
Variations in the levels of propionic and butyric acid (**A**), lactic, pyruvic and formic acid (**B**) and pH (**C**) with respect to values in baseline (time 0) after 24 h of incubation in fecal cultures. *, Significant differences at *p* < 0.05.

Regardless of 2′FL degradation rate or 2′FL commercial preparations used, the fecal microbiota in FF cultures produced more propionic acid while in BF cultures produced more butyric acid (Figure 5). These differences were also accompanied by higher levels of pyruvic and formic acids in fecal cultures of BF infants and by more lactic acid production in cultures of FF infants, which could account for the more pronounced decrease in pH in the FF cultures during incubation (Figure 5C). No significant differences were found due to feeding type in the production of gas and other metabolites analyzed (data not shown).

#### 3.4.3. Effect of 2′FL Fermentation Capacity on the Microbial Metabolic Activity of Fecal Cultures

The microbial enzymatic degradation of 2′FL released primarily fucose and lactose. Fucose could be consumed by microorganisms or accumulate in the culture medium. Lactose, produced either from 2′FL degradation or present in the culture medium, could be enzymatically hydrolyzed by the intestinal microbiota to release glucose and galactose, which can, in turn, accumulate or be used as fermentable carbon sources by microorganisms. Levels of fucose, glucose and lactose in cultures is the result of the net balance between their release from precursor substrates and the subsequent accumulation in the medium or the consumption by microorganisms. Therefore, the concentration of glucose, galactose and fucose were determined as a proxy for the metabolic activity of microorganisms towards 2′FL.

**Figure 6:**
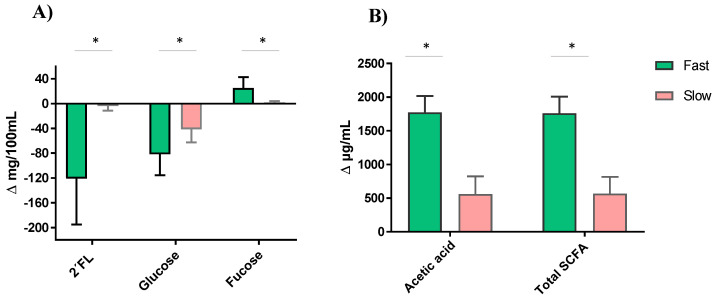
2′FL degradation and variations in the levels of glucose and fucose (**A**), and acetic and total short-chain fatty acids (SCFA), (**B**) with respect to values in baseline (time 0) after 24 h of incubation in fecal cultures with fast and slow fermenting microbiota profiles. *, Significant differences at *p* < 0.05.

Regardless of the type of feeding and the 2′FL commercial preparation used, the fast degraders showed a significantly more pronounced decrease in glucose and a higher accumulation of fucose, which was coincident with a higher production of acetic acid (and total SCFA) (Figure 6). However, there was no difference in galactose accumulation, gas production, pH decreases, BCFA, butyric and propionic acids formation between the fast and slow 2′FL degrader fecal cultures (data not shown).

#### 3.4.4. Combined Effect of 2′FL Fermentation Capacity and Feeding Type on the Microbial Metabolic Activity of Fecal Cultures

The microbial metabolic activity was analyzed separately in fast and slow degrading groups by comparing data variations at 24 h of incubation from cultures inoculated with feces donated by BF and FF babies, added with 2′FL and regardless of the commercial preparation used (Table 2). For 2′FL-fast degraders, cultures with feces from the FF infant presented more pronounced decreases of pH during fermentation, higher increases of succinic and lactic acid, and more glucose consumption than cultures of BF, with no significant differences in the profile of the other parameters analyzed. In contrast, more parameters were affected in slow degrader fecal cultures depending on the donor feeding type. In cultures from the BF infant, more production of gas, isobutyric and butyric acids, higher formation of pyruvic and formic acids and higher consumption of galactose was obtained, whereas in cultures of FF infants occurred a more pronounced decrease of pH, higher production of acetic and lactic acid, and a higher accumulation of galactose and fucose. As a consequence of this, in slow degrading cultures, those from FF donors presented more pronounced increases in acetic and propionic acid, whereas higher increases in isobutyric and butyric acid were observed in cultures of BF infants than in those receiving formula. Moreover, breastfeeding seems to have a more pronounced impact on the profile of microbial metabolites in cultures of slow degraders than in those of fast degraders.

## 4. Discussion

In the present study, batch fecal culture fermentations were performed to assess the effect of 2′FL on the microbiota composition and metabolic activity of full-term vaginally delivered babies at two months of age. This age was decided based on a minimum time of exposure of babies to the different feeding types as to promote early differences on microbiota profile and whenever enough amount of feces from babies can be guaranteed to carry out experiments. The ANKOM RF system for in vitro batch fermentations used in the present study has been previously employed to assess the efficacy of probiotics and prebiotics to modulate microbiota of extreme obese and normal weight adults [22,31]. The BMIF basal medium was previously optimized to allow a balanced short-term growth of intestinal microorganisms from infant feces [20]. The 24 h fermentation time of fecal cultures was chosen as appropriate on the basis of carbon source consumption and microbiota evolution obtained in previous studies [20,22].

Infant feeding mode has been considered one of the main drivers of the early intestinal microbial colonization [32]. Therefore, we initially choose the six donors for our work in an equilibrated way by selecting three exclusively breastfed and three exclusively formula-fed infants, in order to cover the microbiota variability related to the mode of feeding and to generate enough data supporting conclusions obtained from fecal cultures. However, a clear bimodal distribution of fecal inocula was observed as a function of the ability of their microbiotas to degrade 2′FL during in vitro fermentation, segregating donor samples in two groups: fast degraders (more than 90% 2′FL degraded after 24 h of incubation) and slow degraders (less than 5–6% 2′FL degraded after 24 h of incubation). The ability to degrade 2′FL by our fecal inocula appears independent from the mode of feeding, as two FF donors and one BF donor were slow degraders whereas two BF babies and one FF baby were fast degraders; however, due to the low number of infant donors in our study, a clear conclusion in that regard cannot be established. A differential fermentation ability of 2′FL by infant fecal samples during in vitro colon simulations has been previously reported [33] and the consumption of HMOs by newborns during the first days of life has been related to differences in the early composition of the infant fecal microbiota [11,34].

The gut microbiota composition of our fecal inocula showed a large heterogeneity at baseline, in accordance with that reported previously by us and by other authors [1,32,35]. Our results also suggest a different ability to utilize 2′FL among infant donors as a function of the basal microbiota composition. Although bifidobacteria are frequently described as one of the most abundant taxa in breastfed infants, we observed a dominance of Enterobacteriaceae in the six fecal samples, which is in accordance with our previous observations in infants under three months [36]. These differences in dominant taxa could be partly due to the different methodologies used among studies [1]. Streptococci were the second taxa in abundance in samples from the FF-slow degrader donors whereas the Enterococcaceae family was the most abundant in the inoculum of the FF-fast degrader baby and the second in relative abundance in that of the BF-slow degrader baby. Fecal levels of *Streptococcus* and *Enterococcus* have been previously associated with formula feeding type in babies until two–three months of life [37,38]. As expected, bifidobacteria were among the predominant taxa in all inocula except in the sample of the BF-slow degrader baby, where its abundance was strikingly low. At this point, it is interesting to mention that rare cases of bifidobacteria-negative babies have been reported both for those fed human milk or formula [39]. Despite microbiota heterogeneity among samples, the inocula presented clearly differential microbial profiles as a function of donor feeding type and the 2′FL degradation rate in fecal cultures, which determined four distinct groups of microbiota composition at baseline. The alpha diversity of samples from BF babies was lower than that of FF babies, as previously reported [1,32], and for each feeding type the samples from 2′FL-fast degrader donors displayed higher diversity than those from slow degraders. The sequential microbial colonization that occurs in the infant gut after birth starts with intrauterine and vaginal associated taxa, followed by the colonization of skin-derived facultative anaerobic and aerotolerant taxa such as streptococci and Enterobacteriaceae and then by a dominance of bifidobacteriaceae, with the later appearance of adult-like taxa [37].

During the 24 h of incubation in the presence of 2′FL, the microbiota composition of fecal cultures evolved differently depending on the feeding type of donors and the ability of microbiotas to degrade 2′FL. This, together with differences in the intestinal microbiota of newborns that have been reported associated with feeding type or HMO degradation status [40,41] suggest that the so-called “window of opportunity” for the modulation of the microbiota in infants could start very early in life. Among the most abundant microbial taxa, Enterobacteriaceae generally decreased or did not increase significantly in our fecal cultures during incubation. Remarkably, a unique taxon rank, different for each group of cultures, evolved to become dominant or subdominant after 24h of incubation: Lactobacillaceae/*Lactobacillus* group (FF-fast degrader), Streptococcaceae/*Streptococcus* (FF-slow degraders), Bifidobacteriaceae/*Bifidobacterium* (BF-fast degraders). Globally, these results suggest an association of the ability to degrade 2′FL with the increase in bifidobacteria in the fecal cultures of BF babies and with the increase in lactobacilli in those of FF babies. Different studies have shown that some *Bifidobacterium* species, such as *B. bifidum* and *B. longum*, are able to grow using 2′FL as the carbon source [14,18,42,43] whereas others did not. These observations provide support to the high increase of bifidobacteria found in fecal cultures of BF-fast degrader donors where they became the second taxon in abundance after Enterobacteriaceae during fermentation. Levels of lactobacilli have been associated by some authors with high degradation of 2′FL when analyzing feces from BF infants of one month of age [34]. Although lactobacilli have not been proven to ferment efficiently 2′FL in vitro [14,15], they can grow well on metabolites resulting from 2′FL degradation by other microorganisms, through cross-feeding mechanisms [44]. Therefore, 2′FL degradation in fecal cultures of our FF-fast degrader could be associated with the increase in lactobacilli and their interaction with bifidobacteria to consume 2′FL [34]. A reduced ability for degrading 2′FL, probably due to *Bifidobacterium* species not utilizing this HMO, could be the most probable explanation for the slow fermentation of this HMO in fecal cultures of the FF-slow degrader group. In these cultures, the high relative abundance of *Streptococcus* could be related to a higher ability of members of this genus to utilize nutrients available in the culture medium, other than 2′FL, in a competitive way with other members of the fecal microbiota. Finally, in fecal cultures of the BF-slow degrader donor, the low degradation of 2′FL may be due to the scarce presence of 2′ FL-degrading bifidobacteria. At this point, it is interesting to remark that the only FF donor whose fecal cultures degraded 2′FL fast was receiving a formula containing HMO and a probiotic in its composition. The microbiota of this baby showed a clearly different evolution in fecal cultures than that of the other donors. Although the low number of infant donors in this work precludes drawing any conclusion, our results suggest the importance of accomplishing more studies to clarify the influence of different milk formula composition on the establishment of the microbiota of non-breastfed babies, especially regarding the specific probiotics and prebiotics used.

The analysis of the metabolites produced and consumed by the microbiota during the incubation of fecal cultures also revealed differences according to feeding type of donors and 2′FL degradation rate, which reflect differences in metabolic activity and microbial interactions. The production of BCFA has been associated with the colonic microbial fermentation of proteins; a diet rich in non-digestible carbohydrate was shown to decrease the fecal concentration of these compounds in adults [45]. In this regard, we found a decrease in isovaleric acid levels vs. baseline during incubation with 2′FL; This decrease was more pronounced in fecal cultures added with the commercial preparation 2′FL-A, which had the highest purity. As this was the only significant difference in metabolite profiles found between the three commercial preparations used, data for metabolic profiles during fermentation with all three 2′FL formulations were considered jointly in this study.

We found a more pronounced decrease of pH in cultures from FF donors during incubation, which could be related to the higher production of lactic acid. A negative association between pH and lactic and acetic acid levels in infant feces has been previously reported [40,41]. Although bifidobacteria could have contributed, to some extent, to the production of lactic acid in fecal cultures of the FF group, the main microbial producers were probably lactobacilli and streptococci, which were both growing actively in fast and slow fermenter cultures, respectively. In agreement with other authors, we found that the ability to produce acetic acid in fecal cultures, and hence the total levels of SCFA (from which acetic acid is by far the most abundant), was directly related to the ability of the microbiota to ferment 2′FL [33,46]. The capacity for metabolizing 2′FL influences the release and degradation of its constituent monosaccharides, which modifies the availability of fermentable sugars in the culture medium. It seems clear that acetic acid being a major metabolite of Bifidobacterium spp., these microorganisms should have been actively involved in the production of acetic acid during fermentation in cultures of the group of BF-fast degraders, where they became one of the dominant microorganisms during fermentation. Of note, although bifidobacteria were not the dominant microbial group in fecal cultures of the FF-fast degrader donor, the amount of acetic acid produced was similar to that of cultures from BF-fast degrader donors; this supports the metabolic activity of bifidobacteria in such cultures and their interaction with lactobacilli for the efficient degradation of 2′FL, or the involvement of other acetic acid producers.

Lactic and formic acid are generated from pyruvate during glycolysis. The majority of the most abundant taxa in our fecal cultures are able to produce lactic acid by carbohydrate fermentation. However, only some microorganisms of the *Bifidobacterium* genus contribute to the production of formic and lactic acid from HMOs in the infant gut [41], which can in part occur through cross-feeding mechanisms that involve different species or strains participating jointly in the degradation of 2′FL or other HMOs. It has been recently demonstrated that HMO-derived fucose is the preferential route for formic acid production by bifidobacteria in the infant gut whereas lactic acid is mainly synthesized from lactose, and acetic acid could be formed from either substrate [41]. We found a higher pyruvic and formic acid accumulation in fecal cultures of BF donors and a higher concentration of lactic acid in cultures of FF infants. This suggests a possible relationship between the production of formic acid and the feeding type of fecal donors. However, whereas the dominance of bifidobacteria after incubation in fecal cultures from BF-fast degrader group fits well with the preferential production of formic acid by these microorganisms through 2′FL and fucose degradation, the production of formic acid by bifidobacteria cannot be associated with the degradation of 2′FL in cultures from the BF-slow degrader donor. In this case, other sugars present in the medium may have been used by bifidobacteria, promoting a limited growth of this microbial group, which have been even able to accumulate formic acid at higher levels than in the other fecal cultures. In contrast, lactic acid may have been produced by lactobacilli from the lactose released by bifidobacteria from 2′FL in cultures of the FF-fast degrader donor, or by Streptococcaceae or other bacteria from carbohydrates available in cultures of the FF-slow degrader donors.

It has been previously reported that some Enterobacteriaceae could be related to higher succinic acid levels in the infant gut [41,47]. Consistent with these observations, when comparing cultures of fast fermenting donors, we found significantly higher succinic acid levels in FF cultures where Enterobacteriaceae increased during incubation (not significant differences) than in BF cultures where Enterobacteriaceae decreased significantly during fermentation.

The evolution of the less abundant microbial groups during fermentation, some of which are propionic and/or butyric acid producers, could contribute to explain differences found in some fecal cultures for these two SCFA. In this sense, the higher levels of butyric acid in fecal fermentations of the BF group and the higher propionic acid concentration in fecal cultures of the FF group, seems to be determined by the levels of each of these two compounds in the respective cultures of 2′FL-slow degraders. However, just as in cultures of the BF-slow degrader the production of butyric acid was coincident with the increase of butyrate producers from families Ruminococcaceae and Lachnospiraceae (*Blautia*) (not statistically significant) and with a slight decrease of these microorganisms in most of the other fecal cultures during incubation, we cannot hypothesize clearly about the microorganisms that contribute predominantly to propionic acid levels in the cultures of FF-slow degraders.

The ability of intestinal bifidobacteria to utilize 2′FL and the presence of this HMO in breast milk affect the development of the gut microbiota of breastfed infants from very early in life [41,48,49]. The relationship between the maternal secretor status and the colonization of the infant’s gut by 2′FL-utilizing and non-utilizing bifidobacteria is not completely understood yet [40]. Using in vitro fecal cultures from BF and FF infants, which were fast or slow fermenters of 2′FL, we have demonstrated that the effects caused by 2′FL on the gut microbiota of infants differ greatly depending on its composition, which is in turn conditioned by the feeding type and the intrinsic ability of resident bifidobacteria to degrade 2′FL. A limitation of our study is the small sample size (three breastfed and three formula-fed infants), which gives a weak power and does not allow generalizing the results obtained. However, considering the importance of infant’s gut microbiota for the long-term health of individuals, more studies are needed to evaluate the effect of maternal 2′FL on the population of intestinal bifidobacteria utilizing this HMO. When administering 2′FL as prebiotic, the effects could differ among infants. In view of our results and of that from other authors, the characteristics of the individual microbiotas and, specially, of the population of intestinal bifidobacteria should be considered in the development of probiotics and synbiotics containing HMOs.

## 5. Conclusions

The microbiota composition of fecal samples from infants at two months of age was clearly associated with the mode of feeding (breast milk or formula milk) and to its intrinsic ability to degrade 2′FL. The 2′FL degradation rate allowed to segregate fecal cultures as slow and fast degraders, regardless of the feeding type, and determined the evolution of the microbiota during incubation in the presence of this HMO. However, the low number of infant donors in this study does not allow extracting general conclusions. Further studies are needed to understand the role of 2′FL in the colonization of the infant gut and how the early intervention with HMOs could influence the evolution of the microbiota as a function of their differential ability to utilize these molecules.

## Figures and Tables

**Table 1:** Family and genus-level of the most abundant taxa (relative abundance >1% in any of the two taxonomic levels at 0 and/or 24 h of incubation for any of the inocula groups) displaying significant changes during incubation in the presence of 2′FL for the four fecal culture classification groups defined in this study: FF-fast degrader, FF-slow degraders, BF-fast degraders, BF-slow degrader. Relative abundances were compared at baseline and after 24 h of incubation. Significant differences are highlighted in bold (*p*-value < 0.05). Gray shaded cells show relevant changes for the most abundant taxa in each fecal culture.

Taxons	Abundance % (mean ± SD) and Statistical Significance (*p*-Value)											
	FF-Fast Degrader			FF-Slow Degrader			BF-Fast Degrader			BF-Slow Degrader		
	Baseline	24 h	*p*-Value	Baseline	24 h	*p*-Value	Baseline	24 h	*p*-Value	Baseline	24 h	*p*-Value
Bifidobacteriaceae	8.39 ± 2.67	7.78 ± 0.56	0.681	6.56 ± 2.03	8.02 ± 1.27	0.240	10.14 ± 3.41	23.77 ± 8.48	**0.003**	0.03 ± 0.06	2.57 ± 4.23	0.411
*Bifidobacterium*	8.39 ± 2.67	7.77 ± 0.57	0.676	6.56 ± 2.03	8.00 ± 1.29	0.247	10.14 ± 3.41	23.77 ± 8.48	**0.003**	0.03 ± 0.06	2.57 ± 4.23	0.411
Enterobacteriaceae	31.99 ± 6.77	41.29 ± 4.05	0.215	44.34 ± 11.98	33.01 ± 10.60	**0.000**	65.16 ± 19.49	37.36 ± 4.40	**0.007**	72.16 ± 8.00	39.91 ± 15.11	**0.018**
Enterococcaceae	36.01 ± 6.43	3.78 ± 1.00	**0.012**	7.12 ± 7.52	7.96 ± 8.42	0.226	0.34 ± 0.39	0.52 ± 0.58	0.076	6.07 ± 4.36	0.72 ± 0.45	0.182
Erysipelotrichaceae	1.39 ± 0.38	0.18 ± 0.03	**0.037**	0.30 ± 0.47	0.03 ± 0.02	0.203	0.04 ± 0.05	0.01 ± 0.01	0.079	4.02 ± 1.79	2.35 ± 1.35	0.439
Lachnospiraceae	2.57 ± 2.10	0.03 ± 0.03	0.174	1.80 ± 0.82	0.52 ± 0.20	**0.016**	0.34 ± 0.21	0.35 ± 0.25	0.937	0.15 ± 0.06	2.88 ± 4.29	0.388
*Blautia*	0.64 ± 0.57	0.02 ± 0.02	0.204	0.55 ± 0.35	0.14 ± 0.12	**0.022**	0.12 ± 0.12	0.10 ± 0.15	0.818	0.06 ± 0.04	0.64 ± 0.94	0.402
*Lachnospira*	0.28 ± 0.08	0.01 ± 0.01	**0.035**	0.11 ± 0.19	0.01 ± 0.02	0.266	0.03 ± 0.04	0.04 ± 0.05	0.770	0.01 ± 0.02	0.41 ± 0.66	0.397
Lactobacillaceae	0.26 ± 0.13	45.72 ± 5.14	**0.004**	0.48 ± 0.24	8.67 ± 9.09	0.074	0.12 ± 0.13	13.08 ± 14.54	0.079	0.02 ± 0.02	14.99 ± 25.63	0.418
*Lactobacillus* group	0.13 ± 0.04	45.72 ± 5.14	**0.004**	0.34 ± 0.17	8.66 ± 9.09	0.072	0.11 ± 0.13	13.08 ± 14.54	0.079	0.02 ± 0.02	14.98 ± 25.62	0.418
Porphyromonadaceae	0.82 ± 0.61	0.03 ± 0.03	0.162	0.26 ± 0.14	0.05 ± 0.06	**0.008**	0.12 ± 0.10	0.08 ± 0.10	0.590	0.05 ± 0.04	1.30 ± 1.71	0.324
*Parabacteroides*	0.82 ± 0.61	0.03 ± 0.03	0.157	0.26 ± 0.14	0.05 ± 0.06	**0.008**	0.12 ± 0.10	0.08 ± 0.10	0.590	0.05 ± 0.04	1.24 ± 1.61	0.319
Pseudomonadaceae	4.77 ± 2.75	0.24 ± 0.04	0.103	2.94 ± 1.88	0.37 ± 0.12	**0.019**	1.79 ± 1.08	3.30 ± 2.79	0.164	0.61 ± 0.35	3.80 ± 1.76	0.075
*Pseudomonas*	4.77 ± 2.75	0.24 ± 0.04	0.103	2.94 ± 1.88	0.37 ± 0.12	**0.019**	1.79 ± 1.08	3.30 ± 2.79	0.164	0.61 ± 0.35	3.80 ± 1.76	0.075
Ruminococcaceae	0.57 ± 0.79	0.01 ± 0.01	0.343	0.25 ± 0.21	0.02 ± 0.02	**0.038**	0.08 ± 0.08	0.07 ± 0.09	0.784	0.05 ± 0.03	0.72 ± 0.72	0.256
Streptococcaceae	0.44 ± 0.13	0.04 ± 0.01	**0.036**	13.25 ± 4.57	31.26 ± 2.43	**0.000**	0.43 ± 0.14	0.18 ± 0.11	**0.017**	0.44 ± 0.41	0.62 ± 0.83	0.526
*Streptococcus*	0.44 ± 0.13	0.04 ± 0.01	**0.035**	13.22 ± 4.53	31.26 ± 2.43	**0.000**	0.43 ± 0.14	0.17 ± 0.12	**0.013**	0.44 ± 0.41	0.56 ± 0.77	0.614
Veillonellaceae	1.49 ± 0.52	0.07 ± 0.04	**0.041**	6.45 ± 4.36	0.52 ± 0.42	**0.015**	1.26 ± 0.47	0.10 ± 0.04	**0.001**	1.16 ± 0.90	0.08 ± 0.10	0.153
*Veillonella*	1.49 ± 0.52	0.07 ± 0.04	**0.041**	6.40 ± 4.41	0.52 ± 0.42	**0.016**	1.26 ± 0.47	0.10 ± 0.04	**0.001**	1.16 ± 0.90	0.08 ± 0.10	0.153

**Table 2:** Variations from baseline (∆) in the levels of gas, pH, SCFA, organic acids and sugars after 24 h of incubation in fecal cultures with fast and slow fermenting microbiotas according to the feeding type (BF or FF). Significant differences are highlighted in bold (*p*-value < 0.05).

Parameter	2′FL-Fast Degraders			2′FL-Slow Degraders		
	BF	FF	*p*-Value	BF	FF	*p*-Value
**Δ** Gas (mL)	10.99 ± 4.14	11.04 ± 1.55	0.980	11.80 ± 0.31	9.43 ± 1.25	**0.045**
**Δ** pH	−2.49 ± 0.07	−2.68 ± 0.02	**0.003**	−1.80 ± 0.03	−2.49 ± 0.16	**0.000**
**Δ SCFA (µg/mL)**						
Acetic acid	1833.68 ± 230.98	1845.72 ± 318.25	0.949	250.03 ± 32.58	750.99 ± 162.37	**0.001**
Propionic acid	−1.15 ± 1.28	0.00 ± 0.00	0.079	−2.76 ± 2.81	4.92 ± 5.82	0.072
Butyric acid	0.87 ± 2.13	0.00 ± 0.00	0.516	45.72 ± 11.36	0.06 ± 0.14	**0.020**
Isobutyric acid	0.00 ± 0.00	0.41 ± 0.90	0.516	2.80 ± 1.13	−2.24 ± 2.57	**0.016**
Isovaleric acid	−14.34 ± 4.26	−10.79 ± 7.46	0.380	−7.55 ± 8.77	−10.44 ± 7.24	0.613
**Δ Organic acids (mg/100 mL) Δmg/100 mL**						
Lactic acid	223.71 ± 14.88	319.59 ± 16.93	**0.000**	85.80 ± 6.95	247.65 ± 9.81	**0.000**
Succinic acid	9.55 ± 3.40	20.63 ± 1.24	**0.000**	9.54 ± 1.15	9.81 ± 4.66	0.896
Formic acid	6.79 ± 7.35	0.06 ± 0.54	0.075	19.14 ± 5.26	1.76 ± 0.74	**0.028**
Pyruvic acid	1.42 ± 1.14	0.53 ± 0.07	0.116	9.06 ± 1.61	0.19 ± 0.11	**0.011**
**Δ Sugars (mg/100 mL)**						
Lactose	−200.62 ± 43.33	−145.80 ± 26.55	0.089	−30.22 ± 10.87	−196.75 ± 37.07	**0.000**
Galactose	8.92 ± 9.41	−40.66 ± 27.46	0.081	−7.16 ± 2.42	14.38 ± 7.18	**0.000**
Glucose	−61.57 ± 18.45	−128.80 ± 20.59	**0.002**	−29.72 ± 9.18	−47.12 ± 21.14	0.129
Fucose	33.84 ± 10.31	33.28 ± 10.01	0.940	0.05 ± 0.05	2.08 ± 1.29	**0.012**

## Data Availability

The raw sequences reported in this article were deposited in the NCBI Sequence Read Archive (SRA) under the accession number PRJNA731876. Other additional data presented in this study is available upon reasonable request from the corresponding author.
